# Theoretical studies on cycloaddition reactions

**DOI:** 10.1186/1758-2946-6-S1-P2

**Published:** 2014-03-11

**Authors:** Lydia Rhyman, Ponnadurai Ramasami, Luis R Domingo

**Affiliations:** 1 Computational Chemistry Group, Department of Chemistry, Faculty of Science, University of Mauritius, Réduit, Mauritius; 2 Departamento de Química Orgánica, Universidad de Valencia, Dr. Moliner 50, 46100 Burjassot, Valencia, Spain

## 

Cycloaddition reactions represent one of the most powerful processes in organic chemistry. The most common types of cycloaddition reactions are the Diels-Alder (DA) and the 1,3-dipolar cycloaddition reactions (1,3-DCs) which lead to five and six membered rings, respectively.

In our ongoing efforts to contribute to the understanding of DA and 1,3-DCs; we studied the following using the B3LYP/6-31G(d) level of theory:

1. The 1,3-DCs of the pyridinium-3-olates and pyrazinium-3-olates with methyl acrylate and methyl methacrylate [1,2].

2. The competitive hetero-DA and 1,3-DCs of methyl glyoxylate oxime and its tautomeric nitrone with cyclopentadiene in the absence and in the presence of BF_3_ as a Lewis acid catalyst [3].

3. A systematic study on the 1,3-DCs of C_60_ with substituted nitrile oxides (RCNO; R = F, Cl, Br, NC, CN and NO_2_) [4].

Among the outcomes of our investigations is the successful use of theoretical methods to understand the regio- and stereoselectivity of the reactions considered. It is expected that experimentalists find the results useful for synthesis involving these moieties and cycloaddition reactions. This presentation will overview our ongoing research program to have more understanding of these cycloaddition reactions.
